# Brain‐derived neurotrophic factor ( *BDNF*) variants and promoter I methylation are associated with prolonged nocturnal awakenings in older adults

**DOI:** 10.1111/jsr.13838

**Published:** 2023-02-03

**Authors:** Marie‐Laure Ancelin, Isabelle Jaussent, Karen Ritchie, Alain Besset, Joanne Ryan, Yves Dauvilliers

**Affiliations:** ^1^ INM, INSERM Univ Montpellier Montpellier France; ^2^ Institut du Cerveau Trocadéro Paris France; ^3^ Department of Epidemiology and Preventive Medicine Monash University Melbourne Victoria Australia; ^4^ Sleep‐Wake Disorders Unit Department of Neurology, Gui‐de‐Chauliac Hospital CHU Montpellier France

**Keywords:** ageing, biomarker, DNA methylation, epigenetics, sleep

## Abstract

Brain‐derived neurotrophic factor (BDNF) is important for sleep physiology. This study investigates whether *BDNF* variants and promoter I methylation may be implicated in sleep disturbances in older adults. Genotyping was performed for seven *BDNF* single nucleotide polymorphisms (SNPs) in 355 community‐dwelling older adults (aged ≥65 years) and *BDNF* exon 1 promoter methylation was measured in blood samples at baseline (*n* = 153). Self‐reported daytime sleepiness and insomnia, ambulatory polysomnography measures of sleep continuity and architecture, and psychotropic drug intake were assayed during follow‐up. Logistic regression adjusted for age, sex, comorbidities, body mass index, and psychotropic drug intake. Associations were found specifically between wake time after sleep onset (WASO) and four SNPs in the participants not taking psychotropic drugs, whereas in those taking drugs, the associations were either not significant (*rs6265* and *rs7103411)* or in the reverse direction (*rs11030101* and *rs28722151)*. Higher *BDNF* methylation levels were found at most CpG units in those with long WASO and this varied according to psychotropic drug use. The reference group with short WASO not taking drugs showed the lowest methylation levels and the group with long WASO taking treatment, the highest levels. Some SNPs also modified the associations, the participants carrying the low‐risk genotype having the lower methylation levels. This genetic and epigenetic study demonstrated blood *BDNF* promoter methylation to be a potential biomarker of prolonged nocturnal awakenings in older people. Our results suggest the modifying effect of psychotropic drugs and *BDNF* genetic variants in the associations between methylation and WASO.

## 1 INTRODUCTION

Sleep is universal, tightly regulated, and necessary for the proper functioning of neural cells, synaptic connections, and brain circuits. Sleep is key to regulate the total amount of brain synaptic activity (Tononi & Cirelli, 2014). There is synaptic potentiation during the waking day due to ongoing learning, and synaptic down‐scaling during sleep when the brain is disconnected from the environment (Tononi & Cirelli, 2014). This sleep‐dependent weakening of neural activity and synaptic strength is primarily mediated by cortical slow waves. The amount of slow waves gradually decreases with ageing and pathological conditions, although with significant inter‐individual variability (Li et al., 2018). Several other sleep patterns change with ageing, notably advanced sleep pattern and increased frequency of naps and excessive daytime sleepiness (EDS), which is reported in up to 33% of older individuals (Foley et al., 2007). A larger number of nocturnal awakenings and an increase in the time spent awake at night are common in the elderly, particularly in the context of insomnia, also frequent with age (Mander et al., 2017; Riemann et al., 2012). More than one‐third of people aged ≥65 years report symptoms of insomnia (Ohayon & Reynolds 3rd., 2009), and 10%–25% take hypnotics on a regular basis (Jaussent et al., 2013; Ohayon et al., 2012).

Brain‐derived neurotrophic factor (BDNF) is a member of the neurotrophin family of growth factors involved in plasticity of neurones with key roles in the regulation of stress, mood, cognition, metabolism, and sleep. One study reported associations between low levels of serum BDNF and self‐reported insomnia in middle‐aged adults (Giese et al., 2014). However, serum BDNF in middle‐aged adults with insomnia appears to be a biomarker only for insomnia complaints, but not for objectively assessed poor sleep continuity (Mikoteit et al., 2019). The latter study also found a positive association between serum BDNF and the percentage of rapid eye movement (REM) sleep. This association between low serum BDNF and decreased REM sleep has also been reported in another study, but also with the percentage of non‐REM (NREM)3 sleep (Deuschle et al., 2018). Other data showed increased serum BDNF levels with several psychotropic drugs, most commonly being antidepressant REM sleep‐suppressing drugs (Deuschle et al., 2013). Overall, understudied with unclear results, BDNF may be related to sleep continuity and architecture that may contribute to the experience of disrupted and non‐restorative sleep in patients with insomnia.

Several reports have linked *BDNF* genetic variation with circulating BDNF levels (Hing et al., 2018; Januar, Saffery, & Ryan, 2015; Tsai, 2018). In addition, polymorphisms and methylation of the *BDNF* gene have been shown to modulate the availability of the protein in neuropsychiatric disorders (Hing et al., 2018; Januar, Saffery, & Ryan, 2015; Tsai, 2018). However, the studies examining the association between *BDNF* variants and sleep characteristics in the general population are rare and often size limited. They are also mostly focused on adolescents and young adults and psychotropic intake was not considered. To our knowledge, no studies have examined the association of *BDNF* DNA methylation, with self‐reported sleep, polysomnography (PSG) measures, and psychotropic drugs use in older people.

In this study, we investigated whether *BDNF* genetic variants and DNA methylation in community‐dwelling older individuals are associated with self‐reported symptoms of insomnia and objective sleep measures (sleep continuity and architecture) via ambulatory PSG, and whether this may be modified by psychotropic drug intake.

## 2 METHODS

### 2.1 Participants

Data were derived from a longitudinal study of neuropsychiatric disorders in community‐dwelling French older people, the Esprit study (Ritchie et al., 2004). Eligible participants, who were aged ≥65 years and not institutionalised, were recruited by random selection from the electoral rolls between 1999 and 2001. Ethics approval for the study was given by the national ethics committee (Ethical Committee of Sud Méditerranée III and University Hospital of Kremlin‐Bicêtre, France). After obtaining written informed consent from all participants, the study protocol was administered by trained staff at baseline and at up to seven follow‐up waves (with 2–3 year intervals) for 14 years. The study focused on a sample of 355 non‐demented participants, who had complete PSG recordings and provided blood samples, of whom 153 had complete DNA methylation measures.

### 2.2 Sociodemographic and clinical variables

The standardised interview included questions on sociodemographic and behavioural (smoking, alcohol, and caffeine intake) characteristics (Ritchie et al., 2007). Blood pressure, weight, and height were recorded, and body mass index (BMI) calculated (kg/m^2^). Cognitive function was assessed using the Mini‐Mental State Examination (MMSE), a score of ≤26 being considered as cognitive impairment (Folstein et al., 1975). The Center for Epidemiologic Studies Depression scale (CES‐D) was used to assess current depressive symptoms with a score of ≥16 being considered as significant levels of depressive symptomatology (Radloff, 1977). Detailed medical questionnaires were used to obtain information on history of cerebro‐cardiovascular ischaemic pathologies (angina pectoris, myocardial infarction, stroke, cardiovascular surgery, arteritis), as well as other chronic disorders (hypertension, diabetes mellitus, hypercholesterolaemia, thyroid disease, respiratory disease). Dementia was diagnosed by a neurologist as part of a standardised clinical examination and validated by a panel of independent neurologists (Ancelin, Ripoche, et al., 2013). All drugs used in the preceding month were recorded from medical prescriptions and drug packages. Psychotropic drugs affecting sleep were recorded at the time of the PSG examination. They included benzodiazepines, z‐drugs (zolpidem and zopiclone), antidepressants, antihistamines, and miscellaneous medications (including barbiturates, non‐benzodiazepine anxiolytics, and antipsychotics).

### 2.3 Polysomnography measurements and self‐reported sleep problems

Participants underwent, on a voluntary basis, a first night of ambulatory PSG recording. Selection of participants was unrelated to sleep problems. PSG recordings took place in the participant's home using the Deltamed (Natus) coherence system, which includes five electroencephalography leads, right and left electro‐oculograms, electromyography of chin and tibialis anterior muscles, electrocardiogram, nasal cannula/pressure transducer, mouth thermistor, chest and abdominal bands, body position, and pulse oximeter. Total sleep time (TST), sleep efficiency, percentages of sleep stages, wake time after sleep onset (WASO) as well as micro‐arousal, periodic leg movement index per hour of sleep (PLMSi), and the Apnea–Hypopnea Index (AHI) were scored manually according to standard criteria (Iber et al., 2007).

Self‐reported sleep disturbances were assessed by the completion of standardised questionnaires close to PSG (median [range] time since PSG 0.94 [0.02–3.01] years). EDS was assessed by the Epworth Severity Scale, a total score >10 indicating a clinically significant EDS (Johns, 1991). The severity of insomnia symptoms was evaluated using the Insomnia Severity Index (ISI) with higher scores suggesting more severe symptoms (score between 0 and 7: absence of insomnia; between 8 and 14: subthreshold insomnia; between 15 and 28: moderate‐to‐severe insomnia symptoms) (Bastien et al., 2001).

### 2.4 *BDNF* genotyping

*BDNF* genotyping was carried out from buccal DNA by LGC Genomics (Hoddesdon, UK) using the KASP SNP genotyping system (Freeman et al., 2003) as described previously (Ancelin, Carriere, et al., 2013). Genotyping was performed for seven polymorphisms selected to represent variation across the entire *BDNF* gene, namely *rs6265* (Val66Met), *rs11030101*, *rs28722151*, *rs7103411*, *rs962369*, *rs908867*, and *rs1491850* (Januar, Ancelin, et al., 2015). Due to the small number of homozygotes for the minor alleles (<6% for four SNPs), especially when exploring the modifying effect of *BDNF* genotype on the associations between sleep measures and methylation, the minor homozygotes were combined with the heterozygotes for analysis (minor alleles).

### 2.5 *BDNF* exon 1 promoter DNA methylation

Genomic DNA was extracted from blood samples collected at baseline. DNA methylation of the *BNDF* promoter 1 region was measured using the Sequenom EpiTYPER mass‐spectrometry platform according to the manufacturer's instructions (Agena Bioscience, San Diego, California, United States), and as described previously (Januar, Ancelin, et al., 2015). The assay covered the region chr11:27,744,025 ‐ 27,744,279 on the UCSC h19 assembly. Forward and reverse primers were tagged with a 10 bp tag (5′‐AGGAAGAGAG) and 31 bp T7‐promoter sequence (5′‐CAGTAATACGACTCACTATAGGGAGAAGGCT). Using this assay, a total of 11 CpG units were measured across promoter I, corresponding to 16 CpG sites. All samples were assayed in triplicate.

### 2.6 Statistical analysis

Chi‐squared tests were used to compare the distribution of *BDNF* genotypes with those predicted under the Hardy–Weinberg equilibrium. Associations between *BDNF* polymorphisms and sleep characteristics were assessed using logistic regression adjusted for potential confounding factors (age, sex, number of chronic disorders, BMI, and psychotropic drug intake). Interactions were tested using the Wald chi‐square test given by the logistic regression model. Multinomial logistic regression models adjusted for age and sex were used to examine *BDNF* methylation according to WASO and psychotropic drug use. The analysis of WASO levels stratified by *BDNF* variants were performed using Wilcoxon–Mann–Whitney test. The significance level was set at *p* < 0.05. Adjustment for multiple comparisons was carried out using the false discovery rate (FDR) method (Benjamini & Hochberg, 1995). Analyses were performed using the Statistical Analysis System (SAS, version v9.4; SAS Institute Inc., Cary, NC, USA).

## 3 RESULTS

The mean age of participants was 80.1 years of whom 59% were female, 12.6% reported EDS and 11.9% had moderate‐to‐severe insomnia symptoms (Table 1). Regarding PSG characteristics, 48.5% had a TST of <6 h, 76.6% a WASO of >60 min, 23.9% an AHI of ≥15 events/h, and 62.6% a PLMSi ≥15 events/h. As no clinical thresholds exist to determine sleep efficiency, slow‐wave sleep (SWS), and REM sleep, the lowest tertile values (51.5%, 9.2% and 16.3%, respectively) were used as cut‐offs and corresponded to the most severe conditions. High TST, sleep efficiency, SWS and REM sleep as well as low AHI, iPLMS, and WASO were considered as references. More than 37% of the participants were taking at least one psychotropic drug at the time of PSG recording; benzodiazepines (19.7%), z‐drugs (14.1%), antidepressants (11.6%, selective serotonin reuptake inhibitors [SSRIs], *n* = 25; non‐selective monoamine reuptake inhibitors, *n* = three) and other antidepressants (mianserine, *n* = four; tianeptine, *n* = four; venlafaxine, *n* = four; milnacipran, *n* = one), antihistaminic compounds (3.7%) and non‐benzodiazepine anxiolytics (0.3%). Compared to the participants not taking psychotropic drugs, those using drugs were more frequently women, had more insomnia symptoms and a higher level of depressive symptoms, longer TST, sleep efficiency, NREM2, but less REM sleep and a shorter WASO (Table 1). Among the 153 patients with DNA methylation measures, >39% were taking at least one psychotropic drug: benzodiazepines (16.3%), z‐drugs (17.0%), antidepressants (9.8%, SSRIs, *n* = seven; non‐selective monoamine reuptake inhibitors, *n* = one), and other antidepressants (mianserine, *n* = two; tianeptine, *n* = one; venlafaxine, *n* = three; milnacipran, *n* = one), and antihistamine compounds (5.9%).

**TABLE 1:** Characteristics of the 355 participants in the whole sample and according to psychotropic drug use

Variables	Whole sample (*N* = 355)	Taking psychotropic drugs		
		No (*N* = 222)	Yes (*N* = 133)	*p*
Sex, female, *n* (%)	209 (58.9)	116 (52.25)	93 (69.92)	0.001
Age, years^a^, mean (SD)	80.1 (4.1)	80.03 (4.02)	80.30 (4.25)	0.553
Alcohol intake (g/day), *n* (%)				0.113
<12	86 (24.5)	52 (23.74)	34 (25.76)	
12–36	241 (68.7)	147 (67.12)	94 (71.21)	
>36	24 (6.8)	20 (9.13)	4 (3.03)	
Caffeine intake (mg/day), *n* (%)				0.597
≤125	122 (35.3)	77 (35.48)	45 (34.88)	
125–375	186 (53.8)	119 (54.84)	67 (51.94)	
>375	38 (10.9)	21 (9.68)	17 (13.18)	
Smoking status, *n* (%)				0.757
Never	204 (57.5)	128 (57.66)	76 (57.14)	
Past	137 (38.6)	84 (37.84)	53 (39.85)	
Current	14 (3.9)	10 (4.50)	4 (3.01)	
Current depressive symptoms (CES‐D ≥ 16), *n* (%)	74 (24.9)	44 (21.26)	30 (33.33)	0.028
Cognitive impairment (MMSE, ≤26), *n* (%)	50 (14.3)	34 (15.53)	16 (12.31)	0.408
Body mass index, kg/m^2^ ^a^, mean (SD)	24.54 (3.3)	24.60 (3.00)	24.45 (3.76)	0.688
Number of other chronic diseases^b^, *n* (%)				0.446
0	70 (19.9)	44 (20.00)	26 (19.85)	
1	118 (33.6)	79 (35.91)	39 (29.77)	
≥2	163 (46.4)	97 (44.09)	66 (50.38)	
Insomnia severity index (ISI), *n* (%)				<0.0001
0–7 No clinically significant insomnia	168 (57.1)	137 (70.62)	31 (31.00)	
8–14 Subthreshold insomnia	91 (31.0)	46 (23.71)	45 (45.00)	
15–21 Clinical Insomnia (moderate severity)	29 (9.9)	10 (5.15)	19 (19.00)	
22–28 Clinical Insomnia (severe)	6 (2.0)	1 (0.52)	5 (5.00)	
Excessive daytime sleepiness (ESS score >10), *n* (%)	36 (12.6)	22 (11.70)	14 (14.29)	0.533
Total sleep time, min^a^, mean (SD)	363.9 (68.1)	358.26 (65.29)	373.30 (71.79)	0.045
Sleep efficiency, %^a^, mean (SD)	56.2 (10.7)	55.07 (9.89)	57.97 (11.87)	0.015
Stage 1, %^a^, mean (SD)	6.8 (3.8)	7.00 (4.03)	6.45 (3.32)	0.182
Stage 2, %^a^, mean (SD)	62.6 (8.9)	61.54 (8.29)	64.41 (9.50)	0.004
Slow‐wave sleep, %^a^, mean (SD)	12.1 (6.6)	12.38 (6.32)	11.61 (7.14)	0.288
REM sleep, %^a^, mean (SD)	18.50 (6.2)	19.08 (5.93)	17.54 (6.45)	0.024
PLMS during sleep index, events/h^a^, mean (SD)	29.0 (25.6)	30.19 (25.43)	27.00 (25.90)	0.257
PLMS during sleep index (events/h), *n* (%)				0.160
<15	132 (37.4)	77 (35.00)	55 (41.35)	
15–30	87 (24.6)	51 (23.18)	36 (27.07)	
≥30	134 (38.0)	92 (41.82)	42 (31.58)	
AHI, events/h^a^, mean (SD)	10.2 (12.1)	11.14 (12.72)	8.72 (10.77)	0.069
AHI (events/h), *n* (%)				0.290
<15	270 (76.1)	163 (73.42)	107 (80.45)	
15–30	52 (14.6)	35 (15.77)	17 (12.78)	
≥30	33 (9.3)	24 (10.81)	9 (6.77)	
Wake time after sleep onset, min^a^, mean (SD)	112.7 (63.7)	118.12 (63.10)	103.70 (63.95)	0.040
SaO_2_ ^a^, %, mean (SD)	93.7 (1.8)	93.69 (1.74)	93.64 (1.78)	0.788
SaO_2_ <90% duration^a^, min, mean (SD)	15.7 (39.3)	14.92 (37.60)	17.05 (42.05)	0.623

^a^ Continuous variables are expressed as mean (standard deviation [SD]).

^b^ Hypertension, diabetes mellitus, hypercholesterolaemia, thyroid disease, respiratory disease, or cerebro‐cardiovascular ischaemic pathologies (angina pectoris, myocardial infarction, stroke, cardiovascular surgery, or arteritis).

Abbreviations: AHI, Apnea–Hypopnea Index; CES‐D, Center of Epidemiological Studies Depression; ESS, Epworth Sleepiness Scale; MMSE, Mini Mental State Examination; PLMS, periodic leg movements during sleep; REM sleep, rapid eye movement sleep; SaO_2_, average oxygen saturation.

### 3.1 Association between *BDNF* genotypes and sleep characteristics

The frequencies of the seven *BDNF* SNPs were not different from those predicted under Hardy–Weinberg equilibrium (*p* > 0.20 for all SNPs) (data not shown). None of the seven SNPs was significantly associated with self‐reported sleep problems or PSG parameters in adjusted models, except for WASO (Tables S1 and S2). Compared to the participants with a shorter WASO, those with a longer WASO were older and more likely to be men, they reported more frequently EDS and had lower TST, sleep efficiency and SWS, but a longer NREM1 **(**Table S3).

Highly significant interaction effects were found with SNPs and psychotropic drug use for WASO (Table 2) but not for other sleep measures. After stratification, associations were found between WASO and four SNPs (*rs6265*, *rs11030101*, *rs28722151*, and *rs7103411*) in the group not taking psychotropic drugs, whereas in those taking psychotropic drugs, the associations were non‐significant for *rs6265* and *rs7103411* and significant but in the opposite direction for *rs11030101* and *rs28722151*. For these two SNPs, the minor alleles were associated with a >85% decreased risk of long WASO in participants not taking psychotropic drugs, but a 3.8 to 5.3‐fold increased odds in those taking drugs. This was even higher for the participants with subthreshold or clinical insomnia (ISI score >7) taking psychotropic drugs (odds ratio [OR] 19.02, 95% confidence interval [CI] 4.29–84.3, *p* = 0.0001; and OR 8.93, 95% CI 2.44–32.7, *p* = 0.001, respectively). All significant associations remained after FDR correction. The same pattern of associations was found further adjusting for depressive symptomatology or cognitive impairment (data not shown).

**TABLE 2:** The association between brain‐derived neurotrophic factor (*BDNF*) polymorphisms and wake time after sleep onset in the whole sample and according to psychotropic drug use

SNP and genotype	Whole sample					Not taking psychotropic drugs					Taking psychotropic drugs				
	≤60 min	>60 min	OR (95% CI)^a^	*p* ^a^	*p*‐interaction (SNP × psychotropic drugs)	≤60 min	>60 min	OR (95% CI)^a^	*p* ^a^	*p*FDR^b^	≤60 min	>60 min	OR (95% CI)^a^	*p* ^a^	*p*FDR^b^
	*N* = 83, %	*N* = 272, %				*N* = 46, %	*N* = 176, %				*N* = 37, %	*N* = 96, %			
*rs6265* ^c^															
GG	67.50	58.52	1	0.191	0.020	70.45	52.00	1	0.010	0.017	63.89	70.53	1	0.344	0.426
AG/AA	32.50	41.48	1.45 (0.83;2.53)			29.55	48.00	2.82 (1.29;6.18)			36.11	29.47	0.66 (0.28;1.55)		
*rs11030101*															
AA	25.32	27.38	1	0.920	<0.0001	6.67	32.35	1	0.002	0.006	50.00	18.28	1	0.0003	0.002
TA/TT	74.68	72.62	0.97 (0.52;1.79)			93.33	67.65	0.13 (0.03;0.45)			50.00	81.72	5.29 (2.13;13.1)		
*rs28722151*															
CC	25.93	33.59	1	0.302	<0.0001	8.89	39.77	1	0.0003	0.002	47.22	21.98	1	0.003	0.009
GC/GG	74.07	66.41	0.73 (0.41;1.32)			91.11	60.23	0.12 (0.04;0.38)			52.78	78.02	3.83 (1.60;9.15)		
*rs7103411*															
TT	66.67	57.41	1	0.131	0.014	68.89	50.29	1	0.005	0.011	63.89	70.65	1	0.366	0.426
TC/CC	33.33	42.59	1.54 (0.88;2.69)			31.11	49.71	3.07 (1.41;6.66)			36.11	29.35	0.67 (0.28;1.59)		
*rs962369*															
AA	51.25	54.69	1	0.684	0.446	62.22	58.08	1	0.978	0.978	37.14	48.31	1	0.218	0.426
AG/GG	48.75	45.31	0.89 (0.52;1.54)			37.78	41.92	1.01 (0.48;2.13)			62.86	51.69	0.57 (0.24;1.39)		
*rs908867*															
GG	86.42	85.28	1	0.835	0.136	91.11	84.88	1	0.295	0.344	80.56	86.02	1	0.299	0.426
GA/AA	13.58	14.72	1.08 (0.51;2.30)			8.89	15.12	1.88 (0.58;6.09)			19.44	13.98	0.57 (0.20;1.65)		
*rs1491850*															
TT	33.33	29.81	1	0.438	0.207	38.10	28.65	1	0.092	0.129	27.78	31.91	1	0.639	0.639
CT/CC	66.67	70.19	1.25 (0.71;2.22)			61.90	71.35	1.98 (0.89;4.38)			72.22	68.09	0.81 (0.34;1.93)		

^a^ Logistic regression adjusted for sex, age, number of comorbidities, body mass index, and psychotropic drugs (for the whole sample).

^b^ *p* values after FDR correction.

^c^ Methionine allele A, Valine allele G.

Abbreviations: CI, confidence interval; FDR, false discovery rate; SNP, single nucleotide polymorphism; OR, odds ratio.

### 3.2 Associations between baseline blood *BDNF* promoter I methylation and PSG characteristics

**FIGURE 1:**
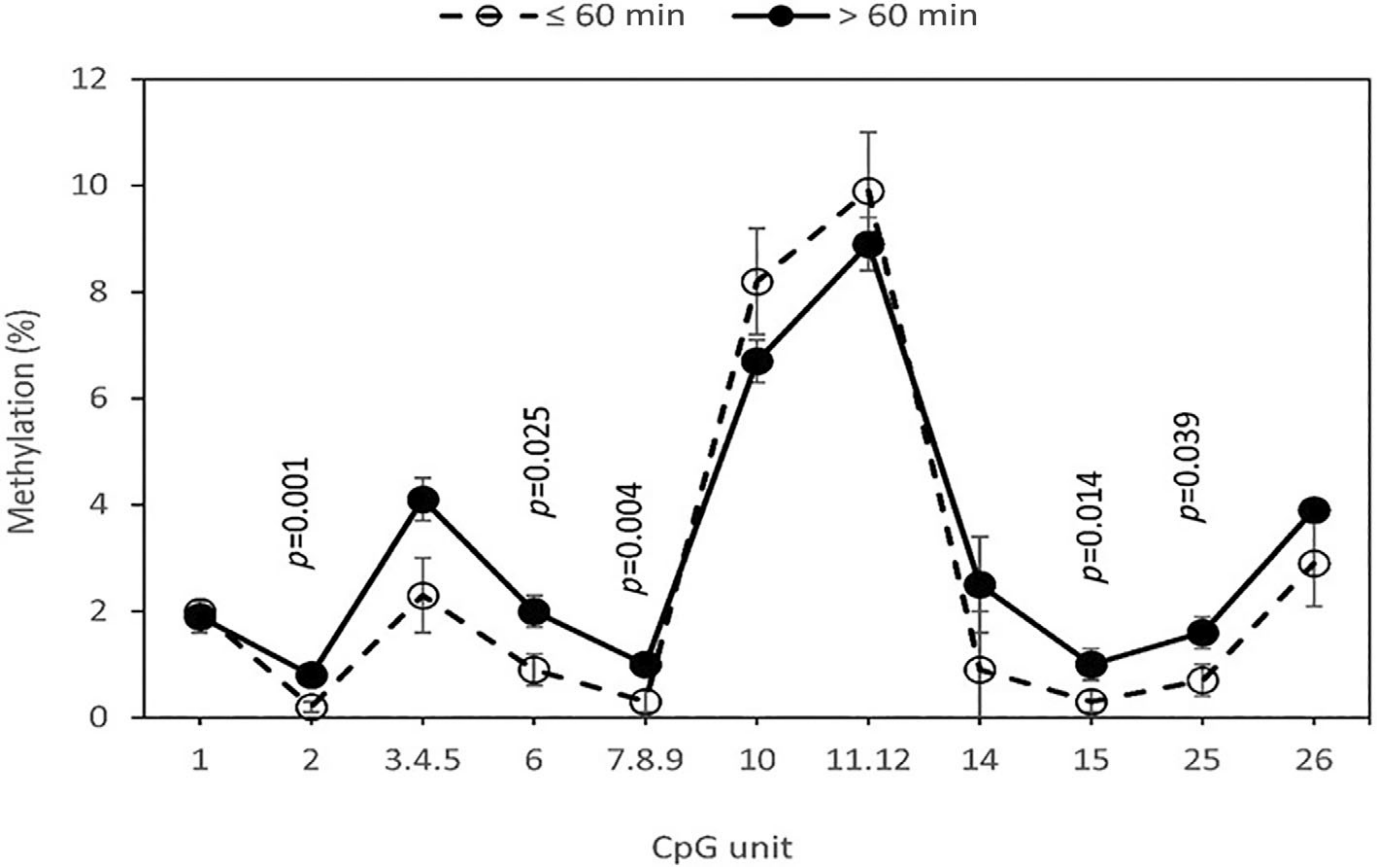
Blood methylation levels in brain‐derived neurotrophic factor (*BDNF*) exon I promoter region in the participants with short (*n* = 33) and long (*n* = 120) wake time after sleep onset (WASO). Data are presented as the geometric mean methylation ± SEM. Dotted lines and open symbols corresponded to the reference group with short WASO (≤ 60 min). Only *p* < 0.05 were indicated.

No significant association was observed between methylation levels and sleep characteristics except WASO (data not shown). Higher *BDNF* methylation levels were observed in the participants with long WASO at several CpG units (Figure 1). The positive associations of WASO with methylation at CpG_2 and CpG_7.8.9 remained significant after FDR correction and corresponded to an increased risk of >30% (OR 1.35, 95% CI 1.13–1.61; and OR 1.34, 95% CI 1.09–1.64, respectively after adjustment for sex, age, and psychotropic drugs). The same pattern of associations was observed when further adjusting for cognitive impairment and depressive symptomatology (data not shown).

The use of psychotropic drugs also modified the association between several CpG units and WASO. We performed a multinomial regression analysis to compare four subgroups of participants, those with short WASO (<60 min) taking or not taking psychotropic drugs, and those with long WASO taking or not taking psychotropic drugs. The participants with short WASO not taking psychotropic drugs (reference group) showed the lowest degree of methylation and those with long WASO taking psychotropic drugs the highest level, whereas the two other groups showed an intermediate pattern (Figure S1). The largest effect size was found at CpG 3.4.5 (Δ = 3.5%). In multivariable analysis, *BDNF* methylation at six CpG units was higher in the participants with long WASO taking psychotropic drugs compared to the reference group (Table 3). This was particularly significant at the CpG_3.4.5 unit with a 4‐fold increased risk. *BDNF* methylation was also higher at CpG_2, CpG_6 and CpG_7.8.9 in those with long WASO not taking psychotropic drugs compared to the reference group. Of those taking psychotropic drugs, *BDNF* methylation at CpG_3.4.5 and CpG_2 was much higher in those with long WASO compared to short WASO.

**TABLE 3:** Multinomial regression model for the association of brain‐derived neurotrophic factor (*BDNF*) methylation levels at six selected CpG units according to wake time after sleep onset and psychotropic drugs.

CpG unit	Global *p*	Short WASO with treatment (*n* = 15) vs. short WASO without treatment (*n* = 18)		Long WASO without treatment (*n* = 75) vs. short WASO without treatment (*n* = 18)		Long WASO with treatment (*n* = 45) vs. short WASO without treatment (*n* = 18)		Long WASO with treatment (*n* = 45) vs. short WASO with treatment (*n* = 15)	
		OR (95% CI)^a^	*p* ^b^	OR (95% CI)^a^	*p* ^b^	OR (95% CI)^a^	*p* ^b^	OR (95% CI)^a^	*p* ^b^
2	0.005	1.21 (0.94;1.56)	0.136	1.38 (1.11;1.72)	0.004	1.64 (1.23;2.18)	0.0008	1.35 (1.00;1.82)	0.049
3.4.5	0.021	1.23 (0.78;1.93)	0.378	1.33 (0.92;1.91)	0.126	4.01 (1.65;9.71)	0.002	3.26 (1.32;8.07)	0.010
6	0.029	1.69 (0.94;3.04)	0.081	1.44 (1.09;1.91)	0.010	1.53 (1.08;2.15)	0.016	0.91 (0.50;1.65)	0.744
7.8.9	0.007	1.39 (0.98;1.97)	0.062	1.47 (1.14;1.88)	0.002	1.59 (1.18;2.15)	0.002	1.14 (0.78;1.67)	0.485
15	0.160	1.03 (0.84;1.28)	0.756	1.11 (0.93;1.32)	0.242	1.25 (1.02;1.54)	0.033	1.21 (0.97;1.50)	0.086
25	0.212	1.14 (0.84;1.54)	0.406	1.15 (0.91;1.45)	0.251	1.43 (1.03;1.99)	0.034	1.26 (0.87;1.81)	0.226

^a^ Adjusted for sex and age.

^b^ Two‐by‐two inter‐group comparisons.

Abbreviations: CI, confidence interval; OR, odds ratio; WASO, wake time after sleep onset.

In this sample, 52% of the participants did not report psychotropic drug use over the follow‐up (never users), 20% taking psychotropic drugs reported having consumed them for more than half of the follow‐ups (chronic users), and 28% were intermittent users. In the subsample from which intermittent users were excluded, the same results were obtained comparing chronic users with never users (data not shown).

### 3.3 *BDNF* promoter I methylation according to WASO, psychotropic drugs and *BDNF* variants

**FIGURE 2:**
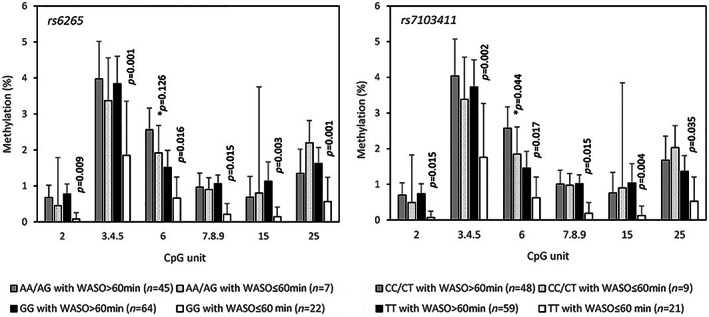
Comparison of in brain‐derived neurotrophic factor (*BDNF*) promoter I methylation at six CpG units in individuals having short or long wake time after sleep onset (WASO), stratified according to *BDNF* genotype (*rs6265* and *rs7103411*). Data are presented as the geometric mean methylation (%) ± SEM. The *p* values calculated from the Wilcoxon–Mann–Whitney test correspond to the comparison between at risk group (1: WASO >60 min) and the reference group (0: WASO ≤60 min) for the carriers of the major homozygotes (*except at CpG_6 where *p* value for minor allele is also reported). *p* > 0.17 for all the other comparisons.

*BDNF* genetic variants associated with WASO also modified the association between WASO and methylation. Lower methylation levels at CpG units_2, 3.4.5, 7.8.9, 15, and 25 were found specifically for the carriers of the major homozygotes of *rs6265* (GG) and *rs7103411* (TT) having short WASO, whereas at CpG_6 this was also observed for the minor allele (Figure 2). Taking into account psychotropic drug use, for *rs6265*, the associations between short WASO and low methylation levels at CpG_2, CpG_6, and CpG_7.8.9 persisted only in the participants not taking psychotropic drugs (*p* ≤ 0.003 compared to *p* > 0.62 in those taking psychotropic drugs) (Figure S2). Conversely, the association at CpG_3.4.5 was significant in both groups, taking (*p* = 0.030) or not (*p* = 0.014) psychotropic drugs. The same pattern was observed for *rs7103411* (data not shown).

## 4 DISCUSSION

To our knowledge, this is the first study in the general population to investigate the role of epigenetic regulation of the *BDNF* gene in sleep, using both self‐reported and objective measures recorded from ambulatory PSG. We found that genetic variation across the *BDNF* gene and promoter I methylation levels were associated with altered sleep continuity in community‐dwelling older adults. Our results also suggest the modifying effect of psychotropic drugs and *BDNF* genetic variants in the association between methylation and WASO. The participants with short WASO not taking psychotropic drugs showed the lowest degree of methylation and those with long WASO taking psychotropic drugs the highest level. Moreover, the functional *rs6265* variant modified these associations, with low methylation level and short WASO found for the carriers of the major homozygotes GG not taking psychotropic drugs.

Several reports have linked *BDNF* genetic variation with circulating BDNF levels (Hing et al., 2018; Januar, Saffery, & Ryan, 2015; Tsai, 2018), as well as blood BDNF levels with sleep disturbances (Furihata et al., 2020; Rahmani et al., 2020; Schmitt et al., 2016). However, the studies examining the association between *BDNF* variants and sleep characteristics in the general population are rare and often size limited. They have focused on the functional variant, Val66Met (*rs6265*), whose minor Met allele (AA+AG) is known to be associated with reduced activity‐dependent secretion of BDNF compared with Val/Val allele (GG). One case–control study reported a greater frequency of heterozygous AG polymorphism in adult patients with insomnia than in a control group, but this was not adjusted for the principal confounders notably depression, BMI, and sex. Psychotropic drug use was also not taken into account (Zaki et al., 2019). In one night laboratory PSG study of 107 middle‐aged and older adults, Gosselin et al. (2016) did not find significant differences between Val/Val and Met carriers in relation to TST, sleep efficiency, number of awakenings, percentage of each sleep stage, REM, and AHI. Also, no significant associations were found in healthy adolescents or younger adults (Grant et al., 2018; Guindalini et al., 2014; Halonen et al., 2019; Halonen et al., 2021). The only study that also examined WASO failed to find significant associations with *BDNF* Val66Met genotypes; however, only 30 healthy young adults were included (Grant et al., 2018).

In the present study, we found that variations across the *BDNF* gene were strongly and specifically associated with WASO. Highly significant associations were found with *rs6265* (2.8‐fold increased risk with Met allele), *rs7103411*, *rs11030101*, and *rs28722151*, in the participants not taking psychotropic drugs, whereas in those taking drugs, the associations were non‐significant with *rs6265* and *rs7103411* or in the opposite direction with *rs11030101* and *rs28722151*. Importantly, these associations were independent of depressive symptoms. This indicates that *BDNF*‐related genetic vulnerability could be linked to WASO phenotype through interaction with psychotropic drugs.

We also found associations between WASO and higher *BDNF* methylation at several CpG sites within promoter I in the participants with long WASO and this varied according to psychotropic drug use. The reference group with short WASO not taking psychotropic drugs showed the lowest methylation levels and the group with long WASO taking treatment showed the highest level, whereas the two other groups showed an intermediate pattern, independently of depression. This was also observed when considering psychotropic treatment duration over the follow‐up. Hence, the association of WASO with *BDNF* variants as well as *BDNF* methylation could be modulated by psychotropic drugs (consisting of benzodiazepines or z‐drugs for 84.7% in our sample), independently of depression. There is some evidence that antidepressants may modify *BDNF* promoter 1 methylation in depression (Hing et al., 2018). Regarding benzodiazepine, acute administration in mice decreased BDNF protein levels within the hippocampus (Licata et al., 2013). Zolpidem significantly reduced exon IV‐ (and marginally exon I‐) containing BDNF transcripts. This was associated with a concomitant increase in the association of methyl‐CpG binding protein 2 (MeCP2) with *BDNF* promoter IV, while also increasing the association of phosphorylated cAMP‐response element binding protein with *BDNF* promoter I (Licata et al., 2013). Further studies are required to determine whether *BDNF* promoter I hypermethylation may represent an underlying mechanism whereby benzodiazepines or z‐drugs reduce BDNF expression in WASO.

In addition, some SNPs including the functional *rs6265* variant, were found to modify the association between WASO and promoter I methylation. The carriers of the low‐risk genotype specifically had lower methylation levels at several CpG units and this was mostly significant in those not taking psychotropic drugs. *BDNF* epigenetic variation has been associated with depression and response to antidepressant medication although not consistently (D. Chen et al., 2017; Hing et al., 2018; Januar, Saffery, & Ryan, 2015; Lisoway et al., 2018). This variable response could be due to sample heterogeneity (differences in population, phenotype, DNA source) or failure to adequately take into account the modifying effect of other factors (e.g., psychotropic medication, genetic variation). We have previously reported interacting effects between *BDNF* variants and buccal promoter I methylation in late‐life depression (Jaussent et al., 2013). Depression was associated with higher methylation for the carriers of the minor allele of *rs6265* and *rs7103411* at CpG_3.4.5 specifically. This differs with the present data on WASO, showing hypomethylation at multiple sites for the carriers of the major homozygotes, independently of depressive symptoms.

The mechanisms by which *BDNF* variants interact with the epigenome remain to be fully elucidated. Genetic variants could influence the probability of DNA methylation, and the location of a SNP in the promoter region, but also at distal sites or intronic regions, may affect how it interacts with the epigenome or phenotype (Januar, Saffery, & Ryan, 2015). DNA methylation can modulate the expression of genes, thus varying the effects driven by individual genetic variants. In the present study, we found that four SNPs including *rs6265* modified the association between WASO and *BDNF* methylation. *Rs6265* is in a protein‐coding region of the gene and may alter BDNF protein function. The Met allele is associated with impaired intracellular trafficking, reduced activity‐dependent secretion of BDNF (Z. Y. Chen et al., 2004), and may affect sleep homeostasis (Halonen et al., 2021). Promoter hypermethylation generally leads to reduced gene expression (Lee et al., 2007). Our findings of elevated *BDNF* promoter methylation associated with abnormal sleep continuity supports observations of reduced circulating BDNF levels in patients with sleep disturbances (Furihata et al., 2020; Rahmani et al., 2020; Schmitt et al., 2016). Disruption in sleep processes may result in or from decreasing BDNF levels, and/or higher stress vulnerability (Schmitt et al., 2016). With advancing age, the impact of *BDNF* variants and promoter methylation are potential factors that could reduce BDNF secretion, brain plasticity, and memory (Gosselin et al., 2016). Overall, the interaction between *BDNF* variants and its promoter methylation, prolonged nocturnal awakenings and the use of psychotropic drugs may regulate brain plasticity in the elderly. However, the precise mechanisms involved, and the temporality of the associations remain to be further elucidated.

This study has several strengths and limitations. This is the first study to investigate the role of *BDNF* epigenetics in a population‐based sample using objective measures of sleep architecture and sleep continuity recorded from ambulatory PSG. Moreover, both genetic and epigenetic variations were considered, as well as the potential modifying effect of confounding factors and psychotropic medication. The genotyping system had a very low error rate, seven SNPs were chosen to ensure satisfactory coverage over the gene and 11 methylation sites were examined in *BDNF* promoter I.

Although the size of our study was relatively large with data on both *BDNF* variants, promoter methylation, and PSG, we were unable in this study to specifically examine *BDNF* homozygotes for the minor alleles due to their low frequencies, methylation differences between brain and blood cells, and the association between psychotropic classes and the effect of monotherapy compared to polytherapy on *BDNF* genetic variants and their methylation levels. Due to the candidate‐based approach, we only focused on the *BDNF* variants. We did not quantify sleep spindles and REM‐sleep fragmentation. DNA methylation signatures were identified in peripheral blood cells, but whether the expression of DNA‐methylation is modified in the same direction in brain remain unknown. We did not measure directly BDNF in the serum.

The lack of repeated evaluations of sleep and DNA methylation precludes establishing causality. The small effect sizes observed should also be considered, as we do not yet know how these could translate into biological differences. However, the changes were found at several CpG sites of *BDNF* promoter I and mostly remained significant after multi‐adjustment and correction for multiple testing. The cumulative effects of such small changes at several sites or over a long period might be expected to result in phenotypic differences large enough to be considered clinically.

In conclusion, our findings indicate that *BDNF* polymorphisms and promoter I methylation are associated with abnormal sleep continuity in older adults. They also suggest the modifying effect of psychotropic drugs and *BDNF* genetic variants in the association between methylation and WASO. Replication in large independent populations and clinical samples is needed to confirm that blood *BDNF* promoter I methylation could be a biomarker of long awakenings at night in the elderly. Further longitudinal studies with repeated phenotyping and biological sample collected at multiple time points to examine the temporal relationship of these associations, may contribute to a better understanding of the pathophysiological mechanisms involved in sleep continuity and the effect of psychotropic drugs.

## AUTHOR CONTRIBUTIONS

Marie Laure Ancelin: study concept and design, data acquisition, result interpretation, preliminary draft writing; Isabelle Jaussent: study concept and design, data acquisition, result interpretation, preliminary draft writing; Karen Ritchie: data acquisition, manuscript revision; Alain Besset: data acquisition, manuscript revision; Joanne Ryan: data acquisition, result interpretation, manuscript revision; Yves Dauvilliers: study concept and design, data acquisition, result interpretation, manuscript revision, and drafting.

## CONFLICT OF INTEREST

Yves Dauvilliers is a consultant for and has participated in advisory boards for Jazz Pharmaceuticals, UCB Pharma, Avadel, Idorsia, Orexia, Takeda, and Bioprojet. The other authors reported no biomedical financial interests or potential conflicts of interest.

## Supporting information


**Data S1.** Supporting Information.

## Data Availability

The data of this study are available upon reasonable request.

## References

[jsr13838-bib-0001] Ancelin, M. L. , Carriere, I. , Scali, J. , Ritchie, K. , Chaudieu, I. , & Ryan, J. (2013). Angiotensin‐converting enzyme gene variants are associated with both cortisol secretion and late‐life depression. Translational Psychiatry, 3, e322. 10.1038/tp.2013.95 24193727 PMC3849962

[jsr13838-bib-0002] Ancelin, M. L. , Ripoche, E. , Dupuy, A. M. , Barberger‐Gateau, P. , Auriacombe, S. , Rouaud, O. , … Ritchie, K. (2013). Sex differences in the associations between lipid levels and incident dementia. Journal of Alzheimer's Disease, 34(2), 519–528. 10.3233/jad-121228 PMC3966213 23254630

[jsr13838-bib-0003] Bastien, C. H. , Vallières, A. , & Morin, C. M. (2001). Validation of the insomnia severity index as an outcome measure for insomnia research. Sleep Medicine, 2(4), 297–307. 10.1016/s1389-9457(00)00065-4 11438246

[jsr13838-bib-0004] Benjamini, Y. , & Hochberg, Y. (1995). Controlling the false discovery rate: A practical and powerful approach to multiple testing. Journal of the Royal Statistical Society. Series B, 57, 289–300.

[jsr13838-bib-0005] Chen, D. , Meng, L. , Pei, F. , Zheng, Y. , & Leng, J. (2017). A review of DNA methylation in depression. Journal of Clinical Neuroscience, 43, 39–46. 10.1016/j.jocn.2017.05.022 28645747

[jsr13838-bib-0006] Chen, Z. Y. , Patel, P. D. , Sant, G. , Meng, C. X. , Teng, K. K. , Hempstead, B. L. , & Lee, F. S. (2004). Variant brain‐derived neurotrophic factor (BDNF) (Met66) alters the intracellular trafficking and activity‐dependent secretion of wild‐type BDNF in neurosecretory cells and cortical neurons. The Journal of Neuroscience, 24(18), 4401–4411. 10.1523/jneurosci.0348-04.2004 15128854 PMC6729450

[jsr13838-bib-0007] Deuschle, M. , Gilles, M. , Scharnholz, B. , Lederbogen, F. , Lang, U. E. , & Hellweg, R. (2013). Changes of serum concentrations of brain‐derived neurotrophic factor (BDNF) during treatment with venlafaxine and mirtazapine: Role of medication and response to treatment. Pharmacopsychiatry, 46(2), 54–58. 10.1055/s-0032-1321908 22961097

[jsr13838-bib-0008] Deuschle, M. , Schredl, M. , Wisch, C. , Schilling, C. , Gilles, M. , Geisel, O. , & Hellweg, R. (2018). Serum brain‐derived neurotrophic factor (BDNF) in sleep‐disordered patients: Relation to sleep stage N3 and rapid eye movement (REM) sleep across diagnostic entities. Journal of Sleep Research, 27(1), 73–77. 10.1111/jsr.12577 28656632

[jsr13838-bib-0009] Foley, D. J. , Vitiello, M. V. , Bliwise, D. L. , Ancoli‐Israel, S. , Monjan, A. A. , & Walsh, J. K. (2007). Frequent napping is associated with excessive daytime sleepiness, depression, pain, and nocturia in older adults: Findings from the National Sleep Foundation '2003 sleep in America' poll. The American Journal of Geriatric Psychiatry, 15(4), 344–350. 10.1097/01.Jgp.0000249385.50101.67 17384317

[jsr13838-bib-0010] Folstein, M. F. , Folstein, S. E. , & McHugh, P. R. (1975). "mini‐mental state". A practical method for grading the cognitive state of patients for the clinician. Journal of Psychiatric Research, 12(3), 189–198. 10.1016/0022-3956(75)90026-6 1202204

[jsr13838-bib-0011] Freeman, B. , Smith, N. , Curtis, C. , Huckett, L. , Mill, J. , & Craig, I. W. (2003). DNA from buccal swabs recruited by mail: Evaluation of storage effects on long‐term stability and suitability for multiplex polymerase chain reaction genotyping. Behavior Genetics, 33(1), 67–72. 12645823 10.1023/a:1021055617738

[jsr13838-bib-0012] Furihata, R. , Saitoh, K. , Otsuki, R. , Murata, S. , Suzuki, M. , Jike, M. , … Uchiyama, M. (2020). Association between reduced serum BDNF levels and insomnia with short sleep duration among female hospital nurses. Sleep Medicine, 68, 167–172. 10.1016/j.sleep.2019.12.011 32044553

[jsr13838-bib-0013] Giese, M. , Unternährer, E. , Hüttig, H. , Beck, J. , Brand, S. , Calabrese, P. , … Eckert, A. (2014). BDNF: An indicator of insomnia? Molecular Psychiatry, 19(2), 151–152. 10.1038/mp.2013.10 23399916 PMC3903111

[jsr13838-bib-0014] Gosselin, N. , De Beaumont, L. , Gagnon, K. , Baril, A. A. , Mongrain, V. , Blais, H. , … Carrier, J. (2016). BDNF Val66Met polymorphism interacts with sleep consolidation to predict ability to create new declarative memories. The Journal of Neuroscience, 36(32), 8390–8398. 10.1523/jneurosci.4432-15.2016 27511011 PMC5811258

[jsr13838-bib-0015] Grant, L. K. , Cain, S. W. , Chang, A. M. , Saxena, R. , Czeisler, C. A. , & Anderson, C. (2018). Impaired cognitive flexibility during sleep deprivation among carriers of the brain derived neurotrophic factor (BDNF) Val66Met allele. Behavioural Brain Research, 338, 51–55. 10.1016/j.bbr.2017.09.025 28947280 PMC5957758

[jsr13838-bib-0016] Guindalini, C. , Mazzotti, D. R. , Castro, L. S. , D'Aurea, C. V. , Andersen, M. L. , Poyares, D. , … Tufik, S. (2014). Brain‐derived neurotrophic factor gene polymorphism predicts interindividual variation in the sleep electroencephalogram. Journal of Neuroscience Research, 92(8), 1018–1023. 10.1002/jnr.23380 24700661

[jsr13838-bib-0017] Halonen, R. , Kuula, L. , Lahti, J. , Makkonen, T. , Räikkönen, K. , & Pesonen, A. K. (2019). BDNF Val66Met polymorphism moderates the association between sleep spindles and overnight visual recognition. Behavioural Brain Research, 375, 112157. 10.1016/j.bbr.2019.112157 31437468

[jsr13838-bib-0018] Halonen, R. , Kuula, L. , Lahti, J. , Räikkönen, K. , & Pesonen, A. K. (2021). The association between sleep‐wake ratio and overnight picture recognition is moderated by BDNF genotype. Neurobiology of Learning and Memory, 177, 107353. 10.1016/j.nlm.2020.107353 33253827

[jsr13838-bib-0019] Hing, B. , Sathyaputri, L. , & Potash, J. B. (2018). A comprehensive review of genetic and epigenetic mechanisms that regulate BDNF expression and function with relevance to major depressive disorder. American Journal of Medical Genetics. Part B, Neuropsychiatric Genetics, 177(2), 143–167. 10.1002/ajmg.b.32616 29243873

[jsr13838-bib-0020] Iber, C. , Ancoli‐Israel, S. , Chesson, A. , & Quan, S. F. (2007). The AASM manual for the scoring of sleep and associated events: Rules, terminology, and technical specifications. Westchester.

[jsr13838-bib-0021] Januar, V. , Ancelin, M. L. , Ritchie, K. , Saffery, R. , & Ryan, J. (2015). BDNF promoter methylation and genetic variation in late‐life depression. Translational Psychiatry, 5, e619. 10.1038/tp.2015.114 26285129 PMC4564567

[jsr13838-bib-0022] Januar, V. , Saffery, R. , & Ryan, J. (2015). Epigenetics and depressive disorders: A review of current progress and future directions. International Journal of Epidemiology, 44(4), 1364–1387. 10.1093/ije/dyu273 25716985

[jsr13838-bib-0023] Jaussent, I. , Ancelin, M. L. , Berr, C. , Pérès, K. , Scali, J. , Besset, A. , … Dauvilliers, Y. (2013). Hypnotics and mortality in an elderly general population: A 12‐year prospective study. BMC Medicine, 11, 212. 10.1186/1741-7015-11-212 24070457 PMC3849429

[jsr13838-bib-0024] Johns, M. W. (1991). A new method for measuring daytime sleepiness: The Epworth sleepiness scale. Sleep, 14(6), 540–545. 10.1093/sleep/14.6.540 1798888

[jsr13838-bib-0025] Lee, B.‐H. , Kim, H. , Park, S.‐H. , & Kim, Y.‐K. (2007). Decreased plasma BDNF level in depressive patients. Journal of Affective Disorders, 101, 239–244. 17173978 10.1016/j.jad.2006.11.005

[jsr13838-bib-0026] Li, J. , Vitiello, M. V. , & Gooneratne, N. S. (2018). Sleep in Normal Aging. Sleep Medicine Clinics, 13(1), 1–11. 10.1016/j.jsmc.2017.09.001 29412976 PMC5841578

[jsr13838-bib-0027] Licata, S. C. , Shinday, N. M. , Huizenga, M. N. , Darnell, S. B. , Sangrey, G. R. , Rudolph, U. , … Sadri‐Vakili, G. (2013). Alterations in brain‐derived neurotrophic factor in the mouse hippocampus following acute but not repeated benzodiazepine treatment. PLoS One, 8(12), e84806. 10.1371/journal.pone.0084806 24367698 PMC3868703

[jsr13838-bib-0028] Lisoway, A. J. , Zai, C. C. , Tiwari, A. K. , & Kennedy, J. L. (2018). DNA methylation and clinical response to antidepressant medication in major depressive disorder: A review and recommendations. Neuroscience Letters, 669, 14–23. 10.1016/j.neulet.2016.12.071 28063933

[jsr13838-bib-0029] Mander, B. A. , Winer, J. R. , & Walker, M. P. (2017). Sleep and Human Aging. Neuron, 94(1), 19–36. 10.1016/j.neuron.2017.02.004 28384471 PMC5810920

[jsr13838-bib-0030] Mikoteit, T. , Brand, S. , Eckert, A. , Holsboer‐Trachsler, E. , & Beck, J. (2019). Brain‐derived neurotrophic factor is a biomarker for subjective insomnia but not objectively assessable poor sleep continuity. Journal of Psychiatric Research, 110, 103–109. 10.1016/j.jpsychires.2018.12.020 30616157

[jsr13838-bib-0031] Ohayon, M. M. , Dauvilliers, Y. , & Reynolds, C. F., 3rd. (2012). Operational definitions and algorithms for excessive sleepiness in the general population: Implications for DSM‐5 nosology. Archives of General Psychiatry, 69(1), 71–79. 10.1001/archgenpsychiatry.2011.1240 22213791 PMC3298734

[jsr13838-bib-0032] Ohayon, M. M. , & Reynolds, C. F., 3rd. (2009). Epidemiological and clinical relevance of insomnia diagnosis algorithms according to the DSM‐IV and the international classification of sleep disorders (ICSD). Sleep Medicine, 10(9), 952–960. 10.1016/j.sleep.2009.07.008 19748312 PMC3715324

[jsr13838-bib-0033] Radloff, L. (1977). The CES‐D scale: A self‐report depression scale for research in the general population. Appl Psychol Measurement, 1, 385–401. 10.1177/014662167700100306

[jsr13838-bib-0034] Rahmani, M. , Rahmani, F. , & Rezaei, N. (2020). The brain‐derived neurotrophic factor: Missing link between sleep deprivation, insomnia, and depression. Neurochemical Research, 45(2), 221–231. 10.1007/s11064-019-02914-1 31782101

[jsr13838-bib-0035] Riemann, D. , Spiegelhalder, K. , Nissen, C. , Hirscher, V. , Baglioni, C. , & Feige, B. (2012). REM sleep instability‐‐a new pathway for insomnia? Pharmacopsychiatry, 45(5), 167–176. 10.1055/s-0031-1299721 22290199

[jsr13838-bib-0036] Ritchie, K. , Artero, S. , Beluche, I. , Ancelin, M. L. , Mann, A. , Dupuy, A. M. , … Boulenger, J. P. (2004). Prevalence of DSM‐IV psychiatric disorder in the French elderly population. The British Journal of Psychiatry, 184, 147–152. 10.1192/bjp.184.2.147 14754827

[jsr13838-bib-0037] Ritchie, K. , Carriere, I. , de Mendonca, A. , Portet, F. , Dartigues, J. F. , Rouaud, O. , … Ancelin, M. L. (2007). The neuroprotective effects of caffeine: A prospective population study (the three City study). Neurology, 69(6), 536–545. 17679672 10.1212/01.wnl.0000266670.35219.0c

[jsr13838-bib-0038] Schmitt, K. , Holsboer‐Trachsler, E. , & Eckert, A. (2016). BDNF in sleep, insomnia, and sleep deprivation. Annals of Medicine, 48(1–2), 42–51. 10.3109/07853890.2015.1131327 26758201

[jsr13838-bib-0039] Tononi, G. , & Cirelli, C. (2014). Sleep and the price of plasticity: From synaptic and cellular homeostasis to memory consolidation and integration. Neuron, 81(1), 12–34. 10.1016/j.neuron.2013.12.025 24411729 PMC3921176

[jsr13838-bib-0040] Tsai, S. J. (2018). Critical issues in BDNF Val66Met genetic studies of neuropsychiatric disorders. Frontiers in Molecular Neuroscience, 11, 156. 10.3389/fnmol.2018.00156 29867348 PMC5962780

[jsr13838-bib-0041] Zaki, N. F. W. , Saleh, E. , Elwasify, M. , Mahmoud, E. , Zaki, J. , Spence, D. W. , … Pandi‐Perumal, S. R. (2019). The association of BDNF gene polymorphism with cognitive impairment in insomnia patients. Progress in Neuro‐Psychopharmacology & Biological Psychiatry, 88, 253–264. 10.1016/j.pnpbp.2018.07.025 30076879

