# Supplementary material for: Brain‐derived neurotrophic factor ( BDNF) variants and promoter I methylation are associated with prolonged nocturnal awakenings in older adults
Source: J Sleep Res. 2023 Feb 3;32(4):e13838. doi: 10.1111/jsr.13838 (PMC10909562; doi:10.1111/jsr.13838)
Supplement: Supplementary file 1 — Data S1. Supporting Information. [file JSR-32-e13838-s001.docx]

**Table S1. Association of *BDNF* polymorphisms with Epworth Sleepiness Scale (ESS) score and Insomnia Severity Index (ISI) score in the whole sample**

|  | **ESS SCORE** | |  |  |  | **ISI SCORE** | |  |  |
| --- | --- | --- | --- | --- | --- | --- | --- | --- | --- |
| SNP and genotype | ≤10  N=250  % | >10  N=36  % | OR  [95% CI] ^a^ | p^a^ |  | ≤7  N=168  % | >7  N=126  % | OR  [95% CI] ^a^ | p^a^ |
| *rs6265* |  |  |  |  |  |  |  |  |  |
| GG | 61.63 | 63.89 | 1 | 0.475 |  | 56.97 | 68.55 | 1 | 0.260 |
| AG/AA | 38.37 | 36.11 | 0.75 [0.34;1.66] |  |  | 43.03 | 31.45 | 0.73 [0.43;1.26] |  |
| *rs11030101* |  |  |  |  |  |  |  |  |  |
| AA | 23.87 | 35.29 | 1 | 0.463 |  | 24.54 | 30.58 | 1 | 0.205 |
| TA/TT | 76.13 | 64.71 | 0.73 [0.31;1.70] |  |  | 75.46 | 69.42 | 0.69 [0.39;1.23] |  |
| *rs28722151* |  |  |  |  |  |  |  |  |  |
| CC | 28.81 | 38.24 | 1 | 0.523 |  | 31.10 | 33.33 | 1 | 0.454 |
| GC/GG | 71.19 | 61.76 | 0.76 [0.33;1.75] |  |  | 68.90 | 66.67 | 0.81 [0.46;1.41] |  |
| *rs7103411* |  |  |  |  |  |  |  |  |  |
| TT | 59.76 | 64.71 | 1 | 0.421 |  | 56.36 | 65.57 | 1 | 0.509 |
| TC/CC | 40.24 | 35.29 | 0.72 [0.32;1.61] |  |  | 43.64 | 34.43 | 0.84 [0.49;1.42] |  |
| *rs962369* |  |  |  |  |  |  |  |  |  |
| AA | 52.32 | 45.71 | 1 | 0.397 |  | 53.16 | 49.17 | 1 | 0.758 |
| AG/GG | 47.68 | 54.29 | 1.40 [0.64;3.08] |  |  | 46.84 | 50.83 | 0.92 [0.54;1.57] |  |
| *rs908867* |  |  |  |  |  |  |  |  |  |
| GG | 86.07 | 82.86 | 1 | 0.787 |  | 85.54 | 85.95 | 1 | 0.797 |
| GA/AA | 13.93 | 17.14 | 1.16 [0.40;3.38] |  |  | 14.46 | 14.05 | 0.91 [0.43;1.90] |  |
| *rs1491850* |  |  |  |  |  |  |  |  |  |
| TT | 31.12 | 27.78 | 1 | 0.753 |  | 30.43 | 30.08 | 1 | 0.899 |
| CT/CC | 68.88 | 72.22 | 1.15 [0.49;2.67] |  |  | 69.57 | 69.92 | 0.96 [0.55;1.69] |  |

^a^ Logistic regression adjusted for sex, age, number of comorbidities, body mass index, and psychotropic drugs

**Table S2. Association between *BDNF* polymorphisms and total sleep time, efficiency, slow-wave sleep and rapid eye movement sleep percentages, apnea-hypopnea index and periodic leg movement index per hour of sleep in the whole sample (N=355)**

|  | **TOTAL SLEEP TIME** | |  |  |  | **SLEEP EFFICIENCY** | | |  |  | **SLOW-WAVE SLEEP** | |  |  |
| --- | --- | --- | --- | --- | --- | --- | --- | --- | --- | --- | --- | --- | --- | --- |
| SNP and genotype | ≥6h  N=183  % | <6h  N=172  % | OR  [95% CI] ^a^ | p^a^ |  | ≥51.5%  N=237  % | <51.5%  N=118  % | OR  [95% CI] ^a^ | p^a^ |  | ≥9.23%  N=237  % | <9.23%  N=118  % | OR  [95% CI] ^a^ | p^a^ |
| *rs6265* |  |  |  |  |  |  |  |  |  |  |  |  |  |  |
| GG | 58.89 | 62.35 | 1 | 0.213 |  | 59.66 | 62.39 | 1 | 0.377 |  | 59.40 | 62.93 | 1 | 0.345 |
| AG/AA | 41.11 | 37.65 | 0.75 [0.48;1.18] |  |  | 40.34 | 37.61 | 0.81 [0.50;1.30] |  |  | 40.60 | 37.07 | 0.79 [0.48;1.29] |  |
| *rs11030101* |  |  |  |  |  |  |  |  |  |  |  |  |  |  |
| AA | 28.74 | 25.00 | 1 | 0.326 |  | 27.63 | 25.44 | 1 | 0.618 |  | 28.00 | 24.79 | 1 | 0.259 |
| TA/TT | 71.26 | 75.00 | 1.29 [0.78;2.14] |  |  | 72.37 | 74.56 | 1.15 [0.67;1.95] |  |  | 72.00 | 75.21 | 1.37 [0.79;2.38] |  |
| *rs28722151* |  |  |  |  |  |  |  |  |  |  |  |  |  |  |
| CC | 33.52 | 29.94 | 1 | 0.307 |  | 32.75 | 29.82 | 1 | 0.502 |  | 32.90 | 29.46 | 1 | 0.321 |
| GC/GG | 66.48 | 70.06 | 1.29 [0.79;2.08] |  |  | 67.25 | 70.18 | 1.19 [0.72;1.97] |  |  | 67.10 | 70.54 | 1.30 [0.77;2.20] |  |
| *rs7103411* |  |  |  |  |  |  |  |  |  |  |  |  |  |  |
| TT | 58.19 | 61.08 | 1 | 0.232 |  | 59.83 | 59.13 | 1 | 0.697 |  | 58.01 | 62.83 | 1 | 0.360 |
| TC/CC | 41.81 | 38.92 | 0.76 [0.48;1.20] |  |  | 40.17 | 40.87 | 0.91 [0.57;1.46] |  |  | 41.99 | 37.17 | 0.79 [0.48;1.30] |  |
| *rs962369* |  |  |  |  |  |  |  |  |  |  |  |  |  |  |
| AA | 52.87 | 54.94 | 1 | 0.943 |  | 52.89 | 55.86 | 1 | 0.940 |  | 53.30 | 55.05 | 1 | 0.895 |
| GA/GG | 47.13 | 45.06 | 0.98 [0.62;1.56] |  |  | 47.11 | 44.14 | 0.98 [0.61;1.59] |  |  | 46.70 | 44.95 | 0.97 [0.59;1.59] |  |
| *rs908867* |  |  |  |  |  |  |  |  |  |  |  |  |  |  |
| GG | 86.11 | 84.94 | 1 | 0.612 |  | 85.34 | 85.96 | 1 | 0.977 |  | 83.62 | 89.47 | 1 | 0.094 |
| GA/AA | 13.89 | 15.06 | 1.18 [0.63;2.20] |  |  | 14.66 | 14.04 | 0.99 [0.51;1.91] |  |  | 16.38 | 10.53 | 0.53 [0.25;1.11] |  |
| *rs1491850* |  |  |  |  |  |  |  |  |  |  |  |  |  |  |
| TT | 27.59 | 33.73 | 1 | 0.110 |  | 29.96 | 31.90 | 1 | 0.610 |  | 30.13 | 31.58 | 1 | 0.923 |
| CT/CC | 72.41 | 66.27 | 0.67 [0.41;1.09] |  |  | 70.04 | 68.10 | 0.88 [0.53;1.45] |  |  | 69.87 | 68.42 | 0.97 [0.58;1.63] |  |

^a^ Logistic regression adjusted for sex, age, number of comorbidities, body mass index, and psychotropic drugs.

**Table S2** (con’t)

|  | **RAPID EYE**  **MOVEMENT SLEEP** | |  |  |  | **APNEA-HYPOPNEA**  **INDEX** | |  | |  | |  |  | | **PERIODIC LEG MOVEMENT INDEX** | | | |  |  | |
| --- | --- | --- | --- | --- | --- | --- | --- | --- | --- | --- | --- | --- | --- | --- | --- | --- | --- | --- | --- | --- | --- |
| SNP and genotype | >16.30%  N=237  % | ≤16.30%  N=118  % | OR  [95% CI] ^a^ | p^a^ |  | <15/h  N=270  % | ≥15/h  N=85  % | | OR  [95% CI] ^a^ | | p^a^ |  | | <15/h  N=132  % | | ≥15/h  N=221  % | OR  [95% CI] ^a^ | p^a^ | | |  |
| *rs6265* |  |  |  |  |  |  |  | |  | |  |  | |  | |  |  |  | | |  |
| GG | 56.60 | 68.70 | 1 | 0.093 |  | 61.80 | 56.63 | | 1 | | 0.593 |  | | 61.83 | | 59.45 | 1 | 0.812 | | |  |
| AG/AA | 43.40 | 31.30 | 0.66 [0.40;1.07] |  |  | 38.20 | 43.37 | | 1.16 [0.68;1.98] | |  |  | | 38.17 | | 40.55 | 1.06 [0.66;1.69] |  | | |  |
| *rs11030101* |  |  |  |  |  |  |  | |  | |  |  | |  | |  |  |  | | |  |
| AA | 25.88 | 28.95 | 1 | 0.462 |  | 27.20 | 25.93 | | 1 | | 0.797 |  | | 29.60 | | 25.58 | 1 | 0.686 | | |  |
| TA/TT | 74.12 | 71.05 | 0.82 [0.49;1.38] |  |  | 72.80 | 74.07 | | 1.08 [0.59;2.00] | |  |  | | 70.40 | | 74.42 | 1.11 [0.66;1.86] |  | | |  |
| *rs28722151* |  |  |  |  |  |  |  | |  | |  |  | |  | |  |  |  | | |  |
| CC | 30.26 | 34.78 | 1 | 0.249 |  | 31.15 | 33.73 | | 1 | | 0.615 |  | | 34.88 | | 30.19 | 1 | 0.559 | | |  |
| GC/GG | 69.74 | 65.22 | 0.75 [0.45;1.23] |  |  | 68.85 | 66.27 | | 0.86 [0.49;1.52] | |  |  | | 65.12 | | 69.81 | 1.16 [0.71;1.88] |  | | |  |
| *rs7103411* |  |  |  |  |  |  |  | |  | |  |  | |  | |  |  |  | | |  |
| TT | 54.78 | 69.30 | 1 | 0.040 ^b^ |  | 60.08 | 58.02 | | 1 | | 0.826 |  | | 60.00 | | 58.99 | 1 | 0.916 | | |  |
| TC/CC | 45.22 | 30.70 | 0.61 [0.37;0.99] |  |  | 39.92 | 41.98 | | 1.06 [0.62;1.83] | |  |  | | 40.00 | | 41.01 | 0.97 [0.61;1.56] |  | | |  |
| *rs962369* |  |  |  |  |  |  |  | |  | |  |  | |  | |  |  |  | | |  |
| AA | 55.56 | 50.45 | 1 | 0.497 |  | 52.33 | 58.97 | | 1 | | 0.839 |  | | 47.15 | | 57.35 | 1 | 0.198 | | |  |
| GA/GG | 44.44 | 49.55 | 1.18 [0.73;1.93] |  |  | 47.67 | 41.03 | | 0.94 [0.54;1.65] | |  |  | | 52.85 | | 42.65 | 0.73 [0.46;1.18] |  | | |  |
| *rs908867* |  |  |  |  |  |  |  | |  | |  |  | |  | |  |  |  | | |  |
| GG | 86.52 | 83.62 | 1 | 0.406 |  | 85.11 | 86.90 | | 1 | | 0.505 |  | | 83.08 | | 86.92 | 1 | 0.342 | | |  |
| GA/AA | 13.48 | 16.38 | 1.31 [0.69;2.47] |  |  | 14.89 | 13.10 | | 0.77 [0.35;1.67] | |  |  | | 16.92 | | 13.08 | 0.74 [0.39;1.38] |  | | |  |
| *rs1491850* |  |  |  |  |  |  |  | |  | |  |  | |  | |  |  |  | | |  |
| TT | 29.82 | 32.17 | 1 | 0.873 |  | 33.21 | 22.22 | | 1 | | 0.012^c^ |  | | 27.20 | | 31.94 | 1 | 0.740 | | |  |
| CT/CC | 70.18 | 67.83 | 0.96 [0.58;1.59] |  |  | 66.79 | 77.78 | | 2.27 [1.20;4.29] | |  |  | | 72.80 | | 68.06 | 0.92 [0.55;1.53] |  | | |  |

^a^ Logistic regression adjusted for sex, age, number of comorbidities, body mass index, and psychotropic drugs.

^b^ p-value = 0.32 after FDR correction

^c^ p-value = 0.08 after FDR correction

**Table S3. Demographic, clinical and polysomnographic characteristics according to wake time after sleep onset**

|  | **Wake time after sleep onset** | |  |
| --- | --- | --- | --- |
|  | ≤60 mn  N=83 | >60 mn  N=272 |  |
| **Variables** | n (%) | n (%) | p |
| Sex, % female | 65 (78.31) | 144 (52.94) | <0.0001 |
| Age, in years ^(1)^ | 78.99 (± 3.91) | 80.48 (± 4.10) | 0.004 |
| Alcohol intake (g/day) |  |  | 0.414 |
| <12 | 22 (26.51) | 64 (23.88) |  |
| 12-36 | 58 (69.88) | 183 (68.28) |  |
| >36 | 3 (3.61) | 21 (7.84) |  |
| Caffeine intake (mg/day) |  |  | 0.907 |
| ≤125 | 28 (34.57) | 94 (35.47) |  |
| 125-375 | 45 (55.56) | 141 (53.21) |  |
| >375 | 8 (9.88) | 30 (11.32) |  |
| Smoking status |  |  | 0.120 |
| Never | 55 (66.27) | 149 (54.78) |  |
| Past | 24 (28.92) | 113 (41.54) |  |
| Current | 4 (4.82) | 10 (3.68) |  |
| Current depressive symptoms^,^ (CES-D≥16) | 15 (22.39) | 59 (25.65) | 0.587 |
| Cognitive impairment (MMSE, ≤26) | 11 (13.58) | 39 (14.55) | 0.827 |
| Body mass index, kg/m^2 (1)^ | 24.54 (± 3.40) | 24.54 (± 3.28) | 0.997 |
| Number of other chronic diseases^(2)^ |  |  | 0.438 |
| 0 | 20 (24.10) | 50 (18.66) |  |
| 1 | 24 (28.92) | 94 (35.07) |  |
| ≥2 | 39 (46.99) | 124 (46.27) |  |
| Current psychotropic drugs, yes | 37 (44.58) | 96 (35.29) | 0.127 |
| Insomnia severity index (ISI) |  |  | 0.848 |
| 0-7 No clinically significant insomnia | 38 (58.46) | 130 (56.77) |  |
| 8-14 Subthreshold insomnia | 18 (27.69) | 73 (31.88) |  |
| 15-21 Clinical Insomnia (moderate severity) | 7 (10.77) | 22 (9.61) |  |
| 22-28 Clinical Insomnia (severe) | 2 (3.08) | 4 (1.75) |  |
| Excessive daytime sleepiness (ESS score>10) | 3 (4.55) | 33 (15.00) | 0.035 |
| Total sleep time (mn)^(1)^ | 401.89 (± 62.06) | 352.30 (± 65.67) | <0.0001 |
| Sleep efficiency (%)^(1)^ | 62.82 (± 10.58) | 54.12 (± 9.97) | <0.0001 |
| Stage 1 (%)^(1)^ | 4.88 (± 2.34) | 7.38 (± 3.95) | <0.0001 |
| Stage 2 (%)^(1)^ | 61.71 (± 8.14) | 62.89 (± 9.06) | 0.290 |
| Slow wave sleep (%)^(1)^ | 13.77 (± 6.27) | 11.58 (± 6.68) | 0.009 |
| REM Sleep (%)^(1)^ | 19.64 (± 5.72) | 18.15 (± 6.27) | 0.056 |
| PLMS during sleep index (/hour)^(1)^ | 27.31 (± 24.64) | 29.51 (± 25.93) | 0.494 |
| PLMS during sleep index (/hour) |  |  | 0.857 |
| <15 | 33 (39.76) | 99 (36.67) |  |
| [15-30[ | 19 (22.89) | 68 (25.19) |  |
| ≥30 | 31 (37.35) | 103 (38.15) |  |
| AHI (/h)^(1)^ | 8.47 (± 9.76) | 10.77 (± 12.66) | 0.131 |
| AHI (/h) |  |  | 0.147 |
| <15 | 68 (81.93) | 202 (74.26) |  |
| [15-30[ | 12 (14.46) | 40 (14.71) |  |
| ≥30 | 3 (3.61) | 30 (11.03) |  |
| Sa02^(1)^ | 93.72 (± 1.75) | 93.65 (± 1.76) | 0.746 |
| Sa02 less than 90% duration^(1)^ | 14.19 (± 41.72) | 16.19 (± 38.58) | 0.686 |

^(1)^ Continuous variables are expressed as mean (standard-deviation); ^(2)^ Hypertension, diabetes mellitus, hypercholesterolemia, thyroid disease, respiratory disease, or cerebro-cardiovascular ischemic pathologies (angina pectoris, myocardial infarction, stroke, cardiovascular surgery, or arteritis).

Abbreviations: AHI = Apnea-Hypopnea Index; CES-D = Center of Epidemiological Studies Depression; ESS = Epworth Sleepiness scale; MMSE = Mini Mental State Examination; PLMS = periodic leg movements during sleep; REM sleep = rapid eye movement sleep; Sa02 = Average oxygen saturation.

**Figure S1.** **Association between WASO and *BDNF* methylation levels according to psychotropic drug use**

Data are presented as the geometric mean methylation (%) ± SEM. Only global p-values <0.10 were indicated.

WASO ≤60 mn not taking psychotropic drugs (n=18, dotted line, open circles ) (reference Group).

WASO ≤60 mn taking psychotropic drugs (n=15, grey circles ).

WASO >60 mn taking psychotropic drugs (n=45, black circles ).

WASO >60 mn not taking psychotropic drugs (n=75, dotted line, grey squares )

**Figure S2. Comparison of *BDNF* promoter methylation at 6 selected CpG units in major homozygotes (GG) of *rs6265* having short or long WASO and according to psychotropic drug use**

Data are presented as the geometric mean methylation (%) ± SEM The p-values calculated from the Wilcoxon-Mann-Whitney test correspond to the comparison between at risk group (1: WASO >60 mn) and the reference group (0: WASO ≤60 mn) for the carriers of the major homozygotes. P-values >0.14 for all the other comparisons.
